# Molecular Property Prediction by Combining LSTM and GAT

**DOI:** 10.3390/biom13030503

**Published:** 2023-03-09

**Authors:** Lei Xu, Shourun Pan, Leiming Xia, Zhen Li

**Affiliations:** College of Computer Science and Technology, Qingdao University, Qingdao 266071, China

**Keywords:** deep learning, molecular representation, artificial intelligence, graph convolutional network

## Abstract

Molecular property prediction is an important direction in computer-aided drug design. In this paper, to fully explore the information from SMILE stings and graph data of molecules, we combined the SALSTM and GAT methods in order to mine the feature information of molecules from sequences and graphs. The embedding atoms are obtained through SALSTM, firstly using SMILES strings, and they are combined with graph node features and fed into the GAT to extract the global molecular representation. At the same time, data augmentation is added to enlarge the training dataset and improve the performance of the model. Finally, to enhance the interpretability of the model, the attention layers of both models are fused together to highlight the key atoms. Comparison with other graph-based and sequence-based methods, for multiple datasets, shows that our method can achieve high prediction accuracy with good generalizability.

## 1. Introduction

Traditional drug design [1] is a time-consuming and costly process. In the early stages of drug design, drug development usually relies on the experience of medicinal chemists for the design and validation of drug molecules. The whole process is very complex and lengthy, and the results are uncontrollable and unpredictable. Computer-aided drug design (CADD) [2] is a new approach that can reduce the time, cost, and risk factors involved in the process of drug design, with help of computer technology. The prediction of molecular properties [3] is an important task in CADD, which also is one of the key tasks in cheminformatics. As the data volume of molecular property prediction becomes larger and larger, how to fully utilize these data to improve the accuracy of prediction has received extensive attention.

Density flooding theory (DFT) [4] is a common computational method for molecular property prediction, however, its computational cost is very high, and takes one hour to calculate the properties of a molecule with only 20 atoms [5]. The success of deep learning (DL) in various fields, including image classification [6], video understanding [7], medical imaging [8], and bioinformatics [9,10], shows that deep learning is a powerful tool for learning features from data and task-relevant prediction. In recent years, with the development of big data in biology [11], chemistry [12], and medicine [13], various deep learning algorithms have been optimized and applied to the field of CADD, which has significantly improved the efficiency of drug design and discovery processes.

**Figure 1:**
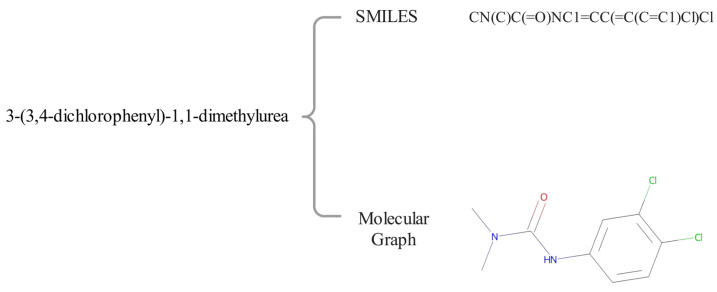
Two representation methods of the same molecule, namely SMILES and molecular graph.

For the molecular property prediction, how to select an appropriate form of molecule is important, and also affects the DL model selection. There are two dominant forms of molecular representation, namely sequences and molecular graphs, as shown in Figure 1. For the sequential representation, the simplified molecular input line entry system [14] (SMILES) is the mostly used method, which encodes molecules into meaningful sequences using certain simple syntactic rules. Recurrent neural networks (RNN) and their variants, such as long- and short-term memory units (LSTM), are widely used to capture features of sequence-based molecular data [15,16]. LSTM units generally consist of input gates, forget gates, output gates, and memory blocks, and they selectively pass information through the gate mechanism by merging the memory units that learn the pattern, in order to forget the previous hidden state and to update the hidden state. Thus, high-level features can be extracted from SMILES for property prediction by LSTM models. In the field of natural language processing (NLP), Word2vec [17] is used to learn high-dimensional embeddings of words. Similarly, Mol2vec [18] is also used to generate embeddings of molecules in the field of biochemistry. Lv et al. [19] mined structural information within atoms and semantic information between atoms using a multilayer LSTM with a hierarchical structure. Datta et al. [20] predicted the lipophilicity of molecules by processing sequences with Mol2vec and LSTM. The model stacked two layers of bidirectional LSTM (BiLSTM), in order to process the molecular substructure. Wu et al. [21] utilized the BiLSTM attention network with a novel multi-step attention mechanism, in order to extract key features from SMILES strings. Zheng et al. [22] proposed a deep learning method, SALSTM, with a self-attention mechanism for processing SMILES, in order to find the relationship between molecular substructures and properties derived from SMILES.

In addition, some studies have applied convolutional neural networks (CNN) to sequence data for molecular feature extraction. For example, Wang et al. [23] used 1D CNN to learn hidden data in sequences, in order to mine deeper information. Oyewola et al. [24] first preprocessed and normalized signal data from molecular properties, then built a 1D CNN to extract the characteristics of the normalized molecular property of the sequence data.

However, there are still a few problems using SMILES to represent a molecule. First, two atoms that are originally adjacent in the molecular structure may be relatively far apart in the sequence. For example, a ring may be broken into a SMILES sequence, in which the connected atoms are the first and last characters in the string. This may affect the accuracy and the performance of the prediction. Second, the SMILES strategy has the problem of non-uniqueness. A molecule may correspond to multiple different SMILES representations. For example, the SMILES of molecule 3-(3,4-dichlorophenyl)-1,1-dimethylurea is CN(C)C(=O)NC1=CC(=C(C=C1)Cl)Cl, which also could be expressed as c1(NC(=O)N(C)C)cc(c(cc1)Cl)Cl or c1(cc(Cl)c(Cl)cc1)NC(=O)N(C)C. This problem also provides a data augmentation method to enhance the performance and generalization of molecular representation. For example, Wu et al. [21] used the SMILES enumeration method in order to increase the number and diversity of training samples. Kimber et al. [25] explored five strategies to augment a single SMILES string into multiple SMILES strings: no augmentation, repeated augmentation, no repeated augmentation, reduced repeated augmentation, and estimated maximum augmentation. They found that the effect of data augmentation is affected by the model and the size of dataset.

Compared with SMILES, molecular graph representation can retain the molecular structure and topological information [26], which can provide richer information for DL models. With the widespread employing of graph neural networks (GNN), which treat atoms and bonds in molecules as nodes and edges, there are many types of GNN used to extract features of graphs, including GCN (graph convolutional networks) [27], GGNN (gated graph neural networks) [28] and DMPNN (directed message passing neural networks) [29]. For the molecular property prediction, Deng et al. [30] proposed the integrated framework XGraphBoost, which addressed the problem of a large number of samples for training in traditional GNN models. Wang et al. [31] proposed a convolutional spatial graph embedding layer (C-SGEL) and a stacked multiple C-SGEL, in order to construct convolutional spatial graph-embedding networks for learning features from molecular graphs.

In addition, the interpretability of a model is also an important aspect, indicating whether the model fits the chemical context, as well as in terms of its representation of key atoms, ascribing certain molecular properties. Weber et al. [32] proposed a simplified and interpretable graph convolutional neural network, which used saliency techniques to highlight molecular substructures associated with the corresponding property. To highlight the importance of specific atoms, Jiménez-Luna et al. [33] proposed the interpretability method, in order to iteratively mask individual atoms and compute molecular fingerprints. In addition, the GraSeq [34] combine both graph encoder and sequence encoder for molecular property.

Although the graph-based [35] and SMILES-based [36] methods for molecular property prediction have developed rapidly, there are still several ways to improve the performance of molecular property prediction.

First, both sequence and graph data provide different views of the molecule, making it important to utilize both of them together to improve the performance. However, just concatenating the information derived from sequence and graph models directly cannot effectively mine the correlation between them. In order to utilize these two forms of molecular representations more effectively, we need to explore a better way to couple these two models more closely, in order to predict molecular properties.

Second, the interpretability of machine learning is one of the most important concerns in this field. For the drug discovery and property prediction, the interpretability is more important, since we need to discover the relationship between the substructure and its property. For property predictions, different functional groups play different roles; thus, how to effectively use the parameters of sequence and graph models to explain the relationship between different functional groups is also an important issue.

Based on this, the main contribution of this paper is as follows: first, in order to fuse the sequence information and graph information of molecules, inspired by GraSeq [34], a method coupling both SALSTM and the graph attention network (GAT) is proposed in this paper. For the same molecule, the information of its SMILES sequence and molecular graph is combined to improve the comprehensiveness and generalization of the molecular representation. Next, to solve the problem of ambiguity in the SMILES sequence, the data augmentation is introduced through the SMILES enumeration method in the preprocessing stage. Finally, attention layers from the SALSTM and GAT methods are grouped together, in order to improve the performance and interpretability of the model. Through the comparative experiments, it is demonstrated that the performance of the proposed model is superior to the SALSTM or GAT model in multiple benchmark datasets.

## 2. Materials and Methods

### 2.1. Overview

**Figure 2:**
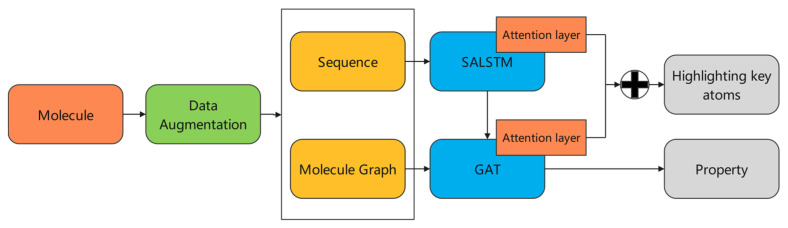
The overall framework.

In this paper, a model incorporating SALSTM and GAT is proposed, combining two strategies in order to improve the performance and interpretability of the model. The overall flow of the model is shown in Figure 2. First, for each molecule, the data augmentation is implemented to generate multiple and non-repetitive SMILES sequences. The corresponding molecular graph is also generated, and, at the same time, the adjacency matrix and the initial feature vector of each atom are obtained based on the molecular graph. Next, the augmented sequence is fed into a SALSTM for feature learning. The feature vector of each atom is updated by the SALSTM, and it is combined with the initial feature vector of atom in the molecular graph as the input of GAT, which ensures that the proposed model combines both the semantic information obtained from SALSTM and the structure information obtained from GAT. Moreover, the attention layer in SALSTM is combined with the attention layer in GAT to highlight the key atoms.

### 2.2. Data Pre-Processing and Augmentation

**Figure 3:**
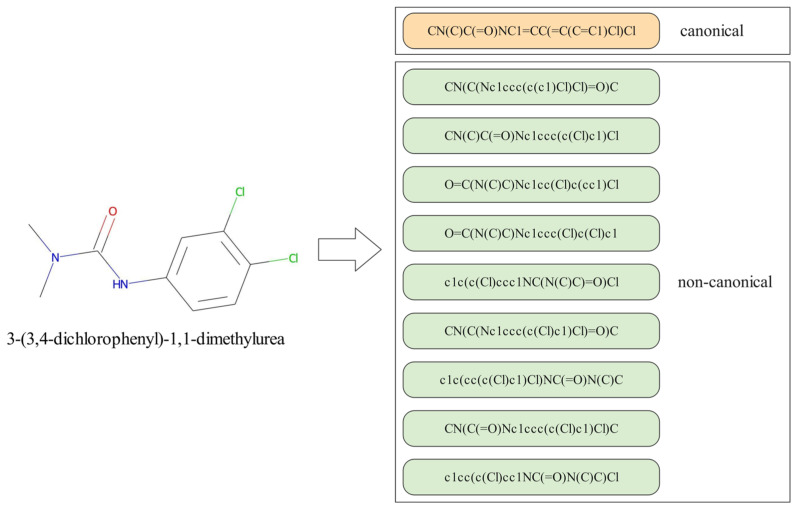
The canonical and non-canonical SMILES representations of the molecule 3-(3,4-dichlorophenyl)-1,1-dimethylurea.

Each molecule corresponds to a unique molecular structure. Due to the ambiguity of the SMILES models, there are multiple SMILES strings that correspond to the same molecule, as shown in Figure 3, which provides an efficient way to enlarge the training dataset to meet the need for DL model training. Specifically, for molecular property prediction tasks, there are two main benefits for using data augmentation. The first one is expanding the datasets and improving the performance and accuracy of model; the second one is improving the generalization of the model to learn the features of the canonical and non-canonical SMILES of the molecule. There are already some methods [37,38,39] that introduce the data augmentation method for molecular property prediction; we used a similar method, with randomly generated SMILES strings.

First, the dataset is split into a training set, a validation set, and a test set in the ratio of 8:1:1. The RDKit chemistry toolkit [40] is used to obtain the canonical SMILES of the input molecule, and its non-canonical SMILES strings are also generated until the number of SMILES strings reach a pre-defined number, which is set as 5 or 10 in this paper according to the different datasets.

Before entering the model, the SMILES string needs to be encoded as a vector for processing. After data augmentation, the dataset is enumerated and each character is marked as a number to build a dictionary, which is same as in the BiLSTM attention network. For some atoms represented by two characters, such as Cl, Br, Si, etc., treating them as two characters will introduce noisy data into the embedding. Instead, the characters R, L, M, etc., are used to represent these atoms. Assuming that the input molecule is a sequence of length N, each atom is represented by a number $X_{n}$.

### 2.3. SALSTM Model

After acquiring the input sequence of each molecule, we first extract the embedding of each atom from the SMILES string by using the SALSTM [22] method.

First, each element of SMILES obtains a corresponding embedding vector through a word embedding algorithm [41], which is then fed into the BiLSTM to obtain the dependencies between neighbor atoms. The BiLSTM is adopted to ensure that each atom could learn the forward and backward information.

In order to improve the interpretability and performance of the whole model, a self-attention mechanism is introduced in the SALSTM, which assigns a corresponding weight to each atom through a set of summation weight vectors for the LSTM hidden states. These multiple attention states are used in SALSTM. In order to simplify the operation, we used a single attention state in this paper. To be specific, the attention mechanism takes the whole BiLSTM hidden state $H\epsilon R^{n^{B}\times d^{B}}$ as an input, and outputs a weight vector $\alpha^{B}$ through the following equation:

(1) $$\alpha^{B}=softmax\left(w_{2}tanh\left(W_{1}H^{T}\right)\right)$$

where $W_{1}$$\epsilon R^{d^{H}\times d^{B}}$ and $w_{2}\epsilon R^{1\times d^{H}}$ denote the weight matrix and vector, respectively, $d^{B}$ is the dimension of the hidden vector of each element, and $n^{B}$ is the number of elements. The attention weight vector $\alpha^{B}$ is expanded to $A^{B}\epsilon R^{n^{B}\times d^{B}}$, and assigned to each element through the following equation:

(2) $$O^{B}=A^{B}⊙H$$

where ⊙ denotes Hadamard products. Since the $O^{B}$ and $A^{B}$ are used as the input for GAT and for interpretability respectively, the features of punctuations and H atoms were removed in $O^{B}$ and $A^{B}$, and the final output of the SALSTM is denoted as $O^{\prime B}\epsilon R^{n\times d^{B}}$ and $A^{\prime B}\epsilon R^{n\times d^{B}}$, where n is the number of atoms of each molecule.

### 2.4. GAT Model

A large number of existing methods focus on using single-sequence-based [42] or graph-based [43,44] models independently for molecular property prediction. However, we believe that combining them together can improve the accuracy of the model. In this paper, the GAT is introduced to process the molecular graph. Moreover, the output $O^{\prime B}$ obtained from the SALSTM is also used as a part of the input of GAT to better learn the local and global structural features of molecules. At the same time, the attention mechanism embedded in the GAT, combined with the attention mechanism in SALSTM, was used to compute the attention scores of the nodes of the molecular graph, in order to obtain the important sub-structure of the graph.

First, we used the RDKit toolkit to obtain the molecular graph, with its adjacency matrix derived from the corresponding SMILES sequence. For each molecular graph, atoms are regarded as nodes and bonds as edges. Atom features are also extracted by the RDKit, and detailed information is shown in Table 1.

**Figure 4:**
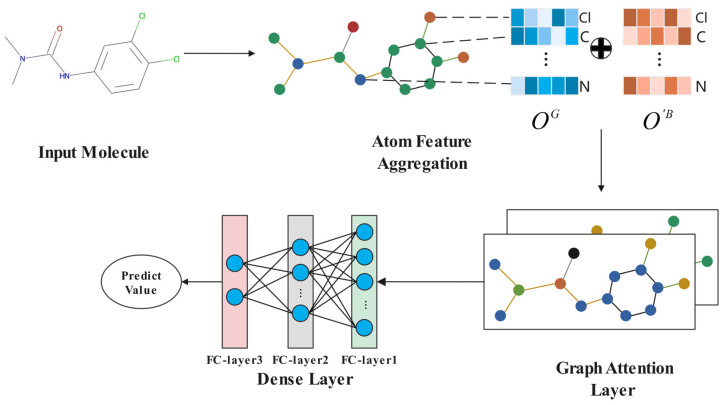
The process of GAT.

The initial feature matrix is $O^{G}\epsilon R^{n\times d^{G}}$, where *n* denotes the number of atoms and $d^{G}$ denotes the initial feature dimension of each atom. The $O^{G}$ is concatenated with $O^{\prime B}$ to obtain the input $I\epsilon R^{n\times \left(d^{B}+d^{G}\right)}$ of GAT. Figure 4 shows the process of the GAT.

The graph attention network [45] is used to extract the final features of molecules, with the help of a self-attention mechanism for each node. Firstly, the attention coefficient $e_{ij}$ is calculated through the following equation:

(3) $$e_{ij}=LeakyReLU(a^{T}\left[WI_{i}\left|\right|WI_{j}]\right),\;\;j\epsilon N_{i}$$

where $e_{ij}$ denotes the attention coefficient of node i with respect to node j, $N_{i}$ denotes the neighborhood of node i, a and W are shared learnable parameters, and || denotes the concatenating operation.

The $e_{ij}$ is fed into a softmax function for normalization.

(4) $$\alpha_{ij}=softmax\left(e_{ij}\right)=\frac{exp\left(e_{ij}\right)}{\sum_{k\epsilon N_{i}}exp\left(e_{ik}\right)}$$

Finally, the node features are updated through aggregating node weight information through the following equation:

(5) $$I_{i}^{\prime }=\sigma \left(\sum_{j\epsilon N_{i}}\alpha_{ij}WI_{j}\right)$$

where σ denotes the nonlinear activation function. $I_{i}^{\prime }$ is a new feature vector of node i after one attention layer.

The weight vectors of each atom i are obtained through the following equation:

(6) $$\alpha_{i}^{G}=\sum_{j\epsilon N_{i}}\alpha_{ij}$$

After GAT processing, molecular features are processed by the dense layer for property prediction. For classification tasks, a sigmoid is used to obtain the final output.

### 2.5. Interpretability

To improve the interpretability of the proposed model, we utilized both attention layers from SALSTM and GAT to highlight the important elements of the molecule. Since the attention scores of two models are different, instead of using a single layer from a sequence-based model or graph-based model, independently, combining them together could help in discovering the importance of atoms, not only in sequence data, but also in terms of molecular structure. In this paper, the attention score of each atom i in the SALSTM attention layer is $a_{i}^{\prime B}$, and the attention score of each atom i in the GAT attention layer is $a_{i}^{G}$; each score is normalized through the min-max normalization as ${\tilde{a}}^{B}$ and ${\tilde{a}}^{G}$, and are added together to obtain the final fusing scores through the following equation:

(7) $$a_{i}={\tilde{a}}_{i}^{B}+{\tilde{a}}_{i}^{G}$$

## 3. Results

### 3.1. Dataset

To fully demonstrate the advantage of the proposed model, several datasets are introduced to evaluate the proposed model in tasks of regression and classification, including ESOL, Lipophilicity, heRG, etc. For each dataset, the data are divided into training, validation, and test sets in a ratio of 8:1:1. Table 2 shows the details of the datasets. ClinTox and Tox21 are multi-classification tasks, in which the input sample corresponds to multiple labels.

#### 3.1.1. Regression Task

For the regression task, we used the root mean square error (RMSE) as the evaluation metric; the lower the RMSE value, the better the prediction performance of the model. We selected three datasets including ESOL, FreeSolv and Lipophilicity datasets, from MoleculeNet (https://moleculenet.org/, accessed on 5 June 2022) [46], and the heRG dataset for the regression task. ESOL dataset [47] contains water solubility data for 1128 compounds. FreeSolv dataset [48] provides experimental and calculated hydration free energies for approximately 642 small molecules in water. Lipophilicity dataset [49] contains 4200 compounds’ experimental results, in terms of their octanol/water distribution coefficient. heRG dataset [50] contains the information of heRG blockers for 6993 compounds.

#### 3.1.2. Classification Task

For the classification task, the ROC–AUC is used as the evaluation metric, and the higher the AUC value, the better the classification performance. We selected three datasets, including the BACE, ClinTox and Tox21 dataset, from MoleculeNet, as well as the Mutagenesis dataset, for the classification task. BACE dataset [51] provides a set of quantitative IC_50 for human β-secretase 1 (BACE-1) inhibitors. ClinTox dataset [52] contains clinical trial toxicity results for 1491 drug compounds. Tox21 dataset [53] contains qualitative toxicity measurements for 7831 compounds across 12 different targets. Mutagenesis dataset [54] contains mutagenesis information for 6506 compounds.

### 3.2. Experiment

In the comparative experiment, we compared the proposed method with various other sequence-based and graph-based approaches, in which FCNN [55], N-GRAM [56], RNNS2S [42], SMILES Transformer [57], FP2VEC [58] and TranGRU [59] are sequence-based model, and SGCN [60], MPNN [61], DMPNN [29], MGCN [62], AttentionFP [63], PreGNN [64], and GraSeq [34] are graph-based models. The results were obtained from public literature and shown in Table 3 and Table 4. The 5-fold cross-validation was used for evaluation.

Compared with other models, our model achieves good results on both regression and classification tasks. In comparison with the previous best results, the improvements are 0.5%, both for the BACE and ClinTox classification task, compared to the best results from N-GRAM and MPNN, respectively. At the same time, the RMSE value of our model decreased by 0.116 in the FreeSolv task compared with that of MPNN.

### 3.3. Ablation Experiment

To further research the factors affecting the performance of the proposed model, several ablation experiments with different parameters were conducted on both classification and regression datasets. For the regression task, we used ESOL, Lipophilicity and heRG datasets for evaluation, since the sizes of these datasets are distributed widely, from 1128 to 4813, which can better explore the differences between datasets. For the classification task, we used BACE, Mutagenesis and Tox21 datasets for evaluation, for the same reason, since the size range of these datasets is from 1513 to 7831. 

#### 3.3.1. Comparison with SALSTM and GAT

**Figure 5:**
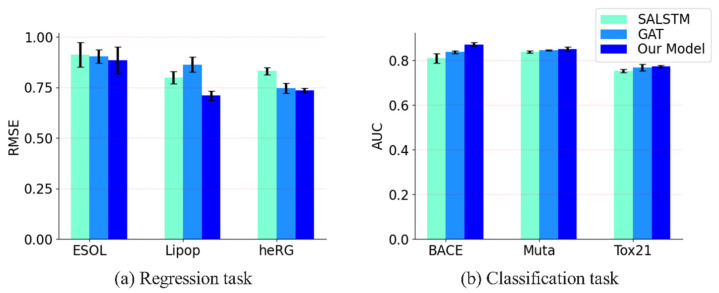
The performance of SALSTM, GAT and our model; the number of augmented samples is 5. (**a**) The performance in the regression task. (**b**) The performance in the classification task. Error bars indicate standard deviation under 5-fold cross-validation.

We combined both SALSTM and GAT together to improve the accuracy of the prediction in this paper. To demonstrate the performance of the proposed model, we compared it with SALSTM and GAT, both in regression and classification tasks, and the results are shown in Figure 5. It can be seen that GAT generally outperforms SALSTM, and after combining both, our model further improved the performance. Figure 5a shows the performance of the three models in the regression task. Compared with SALSTM, our model is improved by 3.2%, 12.5% and 13.0%, and the improvement compared to GAT is 2.1%, 21.7% and 1.6%, respectively. For the classification task, it can be seen from Figure 5b that the proposed method still showed superiority when compared to other methods.

**Figure 6:**
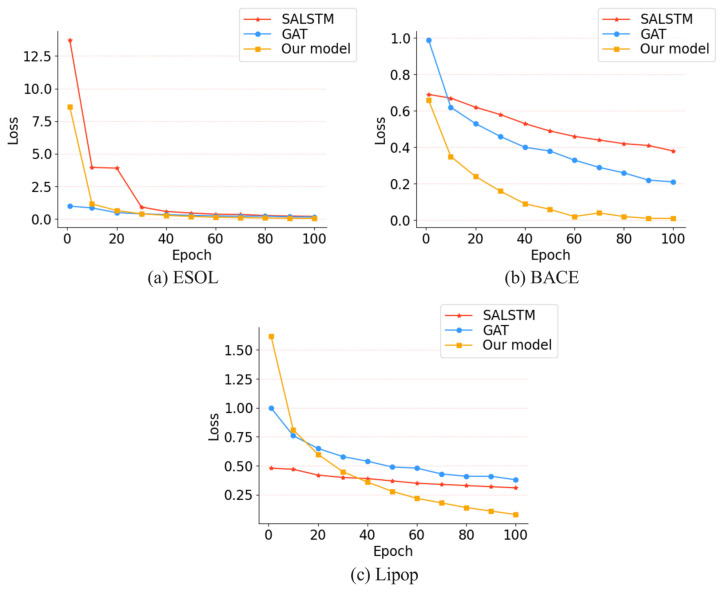
Training loss. (**a**) Training losses of different models for the ESOL dataset. (**b**) Training losses of different models for the BACE dataset. (**c**) Training losses of different models for the Lipop dataset.

Moreover, we compared the differences in training loss of the three methods between the ESOL, BACE and Lipop dataset, as shown in Figure 6. For the ESOL dataset, at the 30th epoch, the losses of our model and GAT are the same. Although the value of loss is similar between our model and GAT, the final loss (epoch 100) of our method is lower than that of GAT. SALSTM is similar to our model in the early stage of training, but the final training loss is higher than GAT and our model. For the BACE dataset, we observed that our model showed a good performance from the early stage of training, the convergence speed is higher than that of GAT and SALSTM, and it tends to be stable at the 60th epoch. For the Lipop dataset, it can be clearly seen from the Figure 6c that, as the number of training rounds increases, our model training loss decreases more significantly than that of SALSTM and GAT. At epoch 40, the loss of our model is lower than that of SALSTM and GAT.

#### 3.3.2. Evaluation on Different Data Augmentation Methods

**Figure 7:**
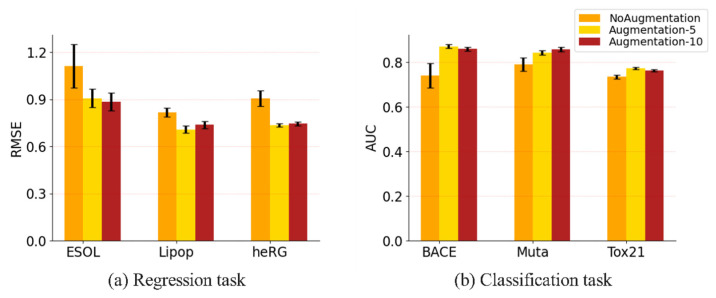
Impact of data augmentation on performance. (**a**) The performance of the regression task. (**b**) The performance of the classification task. Error bars indicate standard deviation under 5-fold cross-validation.

Data augmentation is important in enlarging the training dataset and improving the generalization of the model. To demonstrate the contribution of the data augmentation method, we tested the performance of the model with the augmentation method, including 5 samples and 10 samples, and without the augmentation method; the results are shown in Figure 7. As can be seen from Figure 7a, the model tested with augmentation methods is improved, in terms of the ESOL, Lipophilicity and heRG datasets, when compared to the model tested without augmentation methods. Moreover, the improvements are different, depending on the size of the datasets and the amount of data augmentation. When the number of augmented samples is five, the improvement is about 22.5%, 15.2% and 23.1% for the ESOL, Lipophilicity and heRG datasets, respectively. This shows that data augmentation is very significant for the performance improvement of the model. It can also be seen from Figure 7b that data augmentation improves the model significantly in classification tasks. On the other hand, there is no obvious difference between the methods when using 5 augmented samples and 10 augmented samples. The performance of the model using 10 augmented samples is better than that when using 5 augmented samples, in ESOL and Mutagenesis only, but is worse for the other four datasets. This shows that, in terms of the amount of data augmentation, more is not better. It is possible that the large amount of data augmentation will result in the overfitting of the model, which will affect the performance of the model.

#### 3.3.3. Impact of Adding Attention Mechanism to the Proposed Model

**Figure 8:**
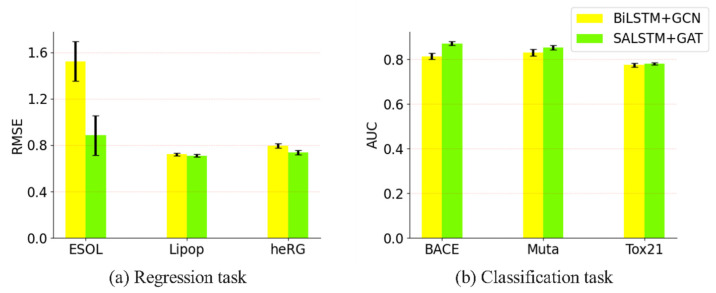
Comparing the effect of attention on the performance of the model. (**a**) The performance of the regression task. (**b**) The performance of the classification task. Error bars indicate standard deviation under 5-fold cross-validation.

We tested the model’s performance with and without the attention mechanism in the regression task and classification task, as shown in Figure 8a,b, respectively. It can be seen that, after adding the attention mechanism, it performed better than when no attention mechanism was added, for the ESOL, Lipophilicity and heRG regression tasks, as well as the BACE, Mutagenesis and Tox21 classification tasks. Through adding the attention mechanism, the molecular embedding obtained is not only semantic information, requiring longer relations, but also structural information between local adjacent atoms.

### 3.4. Interpretability

**Figure 9:**
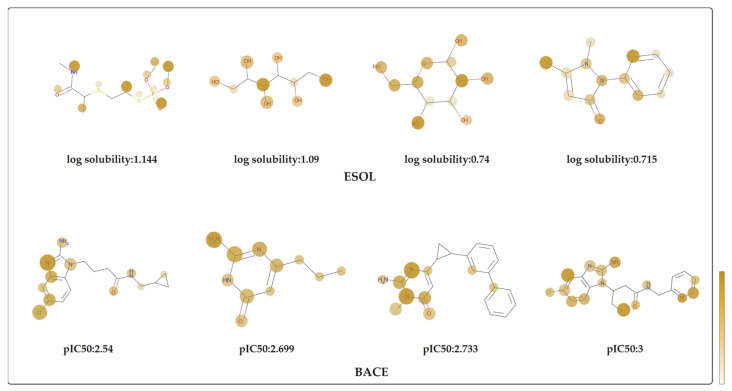
The visualization of molecular attention weights in the ESOL and BACE datasets. The darker the color of the atom, the greater the influence of the atom on the corresponding property.

Besides improving the accuracy of the prediction, the attention mechanism provides an effect way to improve the interpretability of the model. By using the Equation (7) we combined the both attention values from SALSTM and GAT together to highlight the important atoms. We collected the attention weights of the nodes, in order to color each atom of the molecule. The attention weights represent the importance of atoms for the corresponding molecular property, with darker colors representing higher attention weights. Figure 9 shows eight molecules randomly selected from the ESOL and BACE datasets. For ESOL datasets, different functional groups have different importance, in terms of the water solubility of the molecule. It can be obtained from the figure that the functional groups with large attention weights are basically hydrophilic groups, such as hydroxyl(-OH), carbonyl(C=O), etc. This proves that our model can effectively learn molecular representations, and this can provide an effective way for the interpretability of the model. The BACE-1 inhibitors with their attention values are shown in the bottom part of Figure 9. According to the existing studies [65,66], 2-aminoimidazole and isocytosine have inhibitory effects against BACE-1. As shown in the Figure 9, the attention values of the isocytosine or 2-aminoimidazole in the molecule is greater, which is consistent with these studies.

## 4. Conclusions

In this paper, we proposed a model that combines semantic information in molecule sequences and structural information in molecular graphs for predicting molecular properties. Attention mechanisms in the sequence-based model and graph-based model were also fused, in order to learn the key atomic information of the molecule and visualize it. Our model was tested on different datasets, including using regression and classification tasks, and the results demonstrate the generalizability and robustness of the proposed method.

## Figures and Tables

**Table 1:** Atomic Features.

Feature	Description
Atomic number	Atomic number
Degree	Number of directly bonded neighbors (one-hot)
Formal charge	Integer electronic charge (one-hot)
Chiral tag	Chirality information of atoms (one-hot)
Hs num	Number of hydrogen atoms (one-hot)
Hybridization	SP,SP2,SP3,SP3D,SP3D2 (one-hot)
Aromaticity	Whether the atom is in an aromatic hydrocarbon
Mass	Atomic mass

**Table 2:** The details of the datasets.

Dataset	Task	Task Type	#Molecule	Splits	Metric
ESOL	1	Regression	1128	Random	RMSE
FreeSolv	1	Regression	642	Random	RMSE
Lipophilicity	1	Regression	4200	Random	RMSE
heRG	1	Regression	4813	Random	RMSE
BACE	1	Classification	1513	Random	ROC-AUC
Mutagenesis	1	Classification	6506	Random	ROC-ACU
ClinTox	2	Classification	1478	Random	ROC-AUC
Tox21	12	Classification	7831	Random	ROC-AUC

**Table 3:** RMSE scores of regression tasks on test sets. The values in boldface represent the best prediction performances with the corresponding datasets.

		FreeSolv	ESOL	Lipophilicity
Sequence-based	FCNN	1.87 ± 0.07	1.12 ± 0.15	0.86 ± 0.01
	N-GRAM	2.512 ± 0.190	1.100 ± 0.160	0.876 ± 0.033
	RNNS2S	2.987	1.317	1.219
	SMILES Transformers	2.246	1.144	1.169
	FP2VEC	2.512 ± 0.190	1.100 ± 0.160	0.876 ± 0.033
Graph-based	SGCN	2.158 ± 0.049	1.345 ± 0.019	1.074 ± 0.007
	MPNN	1.327 ± 0.279	**0.700 ± 0.073**	0.673 ± 0.038
	DMPNN	2.177	0.980	/
	MGCN	3.349 ± 0.097	1.266 ± 0.147	1.113 ± 0.041
	AttentionFP	2.030 ± 0.420	0.853 ± 0.060	**0.650 ± 0.030**
our method		**1.211 ± 0.192**	0.885 ± 0.067	0.709 ± 0.023

**Table 4:** ROC–AUC scores of classification tasks on test sets. The values in boldface represent the best prediction performances with the corresponding datasets.

		BACE	ClinTox	Tox21
Sequence-based	N-GRAM	0.876 ± 0.035	0.855 ± 0.037	0.769 ± 0.027
	RNNS2S	0.717	\	0.702
	SMILES Transformers	0.719	\	0.706
	TranGRU	0.790	\	0.813
Graph-based	SGCN	\	0.820 ± 0.009	0.766 ± 0.002
	MPNN	0.793 ± 0.031	0.879 ± 0.054	**0.809 ± 0.017**
	MGCN	0.734 ± 0.030	0.634 ± 0.042	0.707 ± 0.016
	AttentionFP	0.863 ± 0.015	0.796 ± 0.005	0.807 ± 0.020
	PreGNN	0.845	**\**	0.781
	GraSeq	0.838	\	0.820
our method		**0.880 ± 0.009**	**0.883 ± 0.025**	0.774 ± 0.005

## Data Availability

Not applicable.
